# Advanced Nanoencapsulated Essential Oil Materials as Novel Antimicrobials for the Control of Fire Blight in Apple

**DOI:** 10.1021/acs.jafc.6c02932

**Published:** 2026-08-01

**Authors:** Andrea Brunelli, Cástor Salgado, José Francisco Fernandez, Nubia Zuverza-Mena, Jason C. White, Quan Zeng

**Affiliations:** † Department of Environmental Sciences, Informatics and Statistics, 117711 Ca’ Foscari University of Venice, Via Torino 155, Venice 30170, Italy; ‡ Instituto de Ceramica y Vidrio (ICV), CSIC, Kelsen 5, 28049 Madrid, Spain; § Department of Analytical Chemistry, The Connecticut Agricultural Experiment Station, New Haven, Connecticut 06511, United States; ∥ Department of Plant Pathology and Ecology, The Connecticut Agricultural Experiment Station, New Haven, Connecticut 06511, United States

**Keywords:** fire blight, Erwinia amylovora, nanobased advanced materials, antibiotic-free disease control, sustainable crop protection

## Abstract

Fire blight, caused by *Erwinia amylovora*, can cause yield losses of up to 95% in apple and pear orchards. Management commonly relies on antibiotic applications during bloom, which may promote antibiotic resistance. Here, bentonite nanoclay (BNT) composites loaded with clove, thymol, or oregano essential oils (EOs) were evaluated as antibiotic-free materials for fire blight control. *In vitro* assays showed that BNT–EO formulations inhibited *E. amylovora* growth, with the minimum inhibitory concentration (MIC) of oregano EO and BNT-oregano (O) estimated at 5 g·L^–1^. Field trials showed that BNT–EO formulations reduced pathogen populations and flower infection over two seasons. In 2023, BNT–O achieved 76% disease reduction, approaching streptomycin efficacy (81%), although efficacy declined in 2024. Elemental analysis confirmed significant enrichment of BNT-derived Al, Na, and Si on BNT–O-treated flowers 9 days postapplication (*p* < 0.001). These findings support EO-loaded nanoclays as promising delivery systems for sustainable fire blight management.

## Graphical abstract

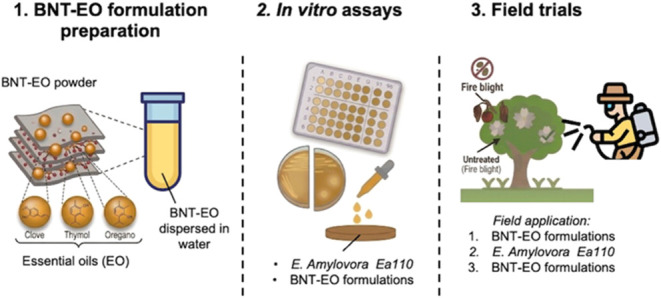

## 1 Introduction

Sustainable agriculture practices aim to ensure long-term food security by balancing environmental stewardship, economic viability, and social well-being. They aim to maintain ecosystem health and biodiversity while sustaining crop productivity and farmer livelihoods through the responsible use of inputs, including a reduced reliance on pesticides. However, this goal is increasingly challenged by persistent plant disease caused by fungi, bacteria, and viruses. Among these, fire blight—caused by the bacterium *Erwinia amylovora*—is one of the most destructive diseases affecting over 130 species of the Rosaceae family, including economically important fruit crops such as apples, pears, medlars, and quinces.^1^ Infection can severely reduce yields and may lead to tree death, resulting in significant ecological and economic impacts. Originally identified in North America, fire blight is now present in more than 40 countries,^2^ across Europe, the Middle East, Asia, North Africa, Central America, and New Zealand,^3^ threatening both cultivated and wild species. Annual losses and management costs exceed USD 100 million in the United States alone,^4^ with significant impacts also reported elsewhere,^5^ including a major outbreak in Switzerland in 2007 that caused approximately USD 56 million in damages and the loss of roughly 40,000 standard trees.^6^

Fire blight infection begins when the pathogen *E. amylovora* infects flowers, where it initially grows on the stigma surface, and then enters hosts through the nectar-secreting glands at the flower hypanthium and causes blossom blight infections. From infected flowers, the pathogen cells further spread to adjacent plant organs through both parenchymatous tissues and the vascular system, reaching nearby shoots, fruit, and stems. As a result, they can lead to shoot blight infection and eventually kill the plant.

All commercially grown apple cultivars are susceptible, leaving growers to rely on chemical controls. Although the use of antibiotic-based agrochemicals (e.g., streptomycin, oxytetracycline, oxolinic acid, and kasugamycin) is restricted in many regions, they remain in use in some countries, such as in the United States, where streptomycin is applied on open flowers. This practice raises concerns due to environmental exposure to materials of clinical importance and the development of resistant pathogens.^7,8^

Among alternative strategies to the use of antibiotics for fire blight management, nonantibiotic biological approaches are being tested. For example, copper-based products, while commonly used, generally provide lower efficacy than streptomycin, sometimes leading to rusting on the fruit surface, which negatively impacts marketability. Other options include exploiting biological approaches, such as beneficial bacteria,^9^ yeasts,^10^ or bacteriophage-based products;^11^ however, the efficacy of these biocontrol agents is often variable under field conditions, and their performance may be less consistent than that of conventional antibiotics, which has so far limited their widespread adoption as complete replacements.

In parallel, extensive research over the past decade has been focused on the effectiveness of plant essential oils (EOs) as antimicrobial agents against resistant bacterial pathogens,^12^ including fire blight, primarily through *in vitro*, *ex vivo*, and/or *in planta* testing.^13–21^ The antimicrobial potential of EOs is ascribed to their complex mixture of volatile compounds, including terpenes, terpenoids, and phenolic constituents (e.g., thymol, carvacrol, and eugenol). However, translating the promising *in vitro* results to field conditions remains challenging due to the susceptibility of active ingredients (AIs) to oxidation, UV radiation, and temperature variations. Commercial EO-based products (e.g., Thyme Guard) are formulated to improve persistence but remain less effective than streptomycin, largely due to rapid volatilization and short residual activity.^22^ As a result, their adoption has been limited, highlighting the need for strategies that enhance EO stability and performance while maintaining environmental sustainability. In this context, the transition toward sustainable agriculture increasingly depends on technological innovation. For example, innovative formulations based on advanced materials (AdMa)—engineered materials designed to impart new functionalities (e.g., stimuli-responsive or targeted delivery) or enhance existing properties such as stability, adhesion, and controlled release^23,24^ —such as nano encapsulation, layered inorganic carriers, and cyclodextrin-based inclusion complexes,^25^ have been shown to improve formulation stability, controlled release, and bioavailability of active compounds, thereby enhancing their performance under field conditions. By encapsulating AI within polymeric or inorganic nanocarriers, these systems protect AIs against premature degradation from UV radiation, heat, moisture fluctuation, and desiccation, while improving solubility, adhesion, and controlled release behavior.^26^ For instance, nanoencapsulated pesticides have been shown to maintain efficacy over extended durations by preventing environmental breakdown and reducing volatilization or leaching.^27^ Similarly, carriers based on layered double hydroxides (LDHs) or cyclodextrin derivatives can protect labile compounds from photolysis and oxidation while enabling sustained or stimulus-responsive release, thereby increasing bioavailability and reducing the required dosage.^25^ Overall, by enhancing the chemical stability, environmental resilience, and delivery efficiency of pesticides, AdMa formulations hold considerable potential to improve disease and pest management in agricultural systems, making protection more consistent, longer-lasting, and potentially more environmentally sustainable.

In one of our previous studies,^28^ a preliminary safety and sustainability assessment of two nanoclay–EO AdMa as antipest systems in light-density polyethylene (LDPE) food packaging was described. The materials consisted of nanodrops of food-grade EO anchored onto nanoclays—either E-558 bentonite (BNT) (layered nanoclay) or E-562 sepiolite (fibrillary nanoclay)—which were subsequently encapsulated with fumaric acid (E-297) and incorporated into an LDPE matrix during thermoforming. This composite served as a sustained-release reservoir, preserving the bioactive properties of the EO while enhancing its stability and compatibility for incorporation into polymeric packaging systems designed for food protection. The screening-level life cycle assessment showed that EO–clay-based materials exhibited a higher share of positive contributions—i.e., outcomes that enhance safety and/or sustainability, as opposed to negative contributions indicating potential risks or adverse impacts—across most life-cycle stages (raw material acquisition, production, manufacturing of the product incorporating the materials, use phase, and end-of-life) compared with conventional LDPE. Although some uncertainty was identified at the raw material stage, the integrated results supported the strategic value of further development and scale-up of these materials within a Safe and Sustainable by Design (SSbD) framework. Of the two nanoclay–EO AdMa, a study was then conducted specifically on the bentonite (BNT)-based material embedded in LDPE to evaluate potential worker and consumer exposure across the life-cycle stages of material production.^29^ The resulting active packaging material demonstrated excellent efficacy in repelling beetles, thereby helping to protect stored or packaged food from pest damage.

In the present study, the application of the AdMa originally developed for food packaging has been extended to the agricultural sector. Given the well-established antimicrobial properties of EOs, our approach was designed to combine the antimicrobial activity of EOs with the physicochemical properties of clay-based carriers, with the aim of improving active-compound delivery, stability, and persistence on plant tissues and thereby enhancing efficacy against plant bacterial diseases, such as fire blight. Accordingly, we tested: (i) the *in vitro* antimicrobial efficacy of different BNT–EO AdMa formulations against the fire blight pathogen *E. amylovora*; (ii) their efficacy in reducing fire blight under the field conditions. The effectiveness of these AdMa highlights their potential to reduce/replace the use of antibiotics in agriculture, supporting more sustainable and environmentally responsible crop protection strategies.

## 2 Materials and Methods

### 2.1 Nanoclays–EO AdMa Synthesis

Synthesis of the different nanoclays–EO AdMa followed a similar procedure to Brunelli et al.^29^ Bentonite (BNT) with a layered platelet morphology (1.6 nm thickness, ca. 600 nm length) was purchased from SEPIOLSA (Minersa Group, Spain; origin: Turkey). The material is a sodium montmorillonite supplied as a fine powder, with a specific surface area of 82 m^2^·g^–1^ and a cation exchange capacity of 86 mequiv·100 g^–1^. Its chemical composition, determined by X-ray fluorescence (fused-bead method), is SiO_2_ (59%), Al_2_O_3_ (14%), MgO (3.4%), Fe_2_O_3_ (2%), CaO (2.3%), and Na_2_O (0.6%). The difference to 100% is primarily attributed to loss on ignition during fused-bead preparation at 1000 °C (17.3%), which is associated with the release of adsorbed water, dehydroxylation of the smectite structure, and the decomposition of minor carbonate phases. The EOs used to generate the BNT–EO AdMa were clove, oregano, and thymol EOs, all purchased from Arocival (Aromes Citrics Valencians S.L.). All of the other reagents and chemicals used were of analytical reagent grade (Merck, KGaA, Darmstadt, Germany). The synthesis of BNT–EO AdMa started with 411 g of raw BNT and 8.4 g of anhydrous sodium carbonate (Na_2_CO_3_), which were separately dispersed in individual containers, each containing 2.9 L of distilled water. Both suspensions were independently mixed with a hand blender for 10 min and let stand for 24 h. Afterward, the suspensions were stirred with a Cowles-type mixer at 1200 rpm for 20 min and filtered with a 100 μm sieve. Both slurries were magnetically filtered and then transferred together to a single 15 L pot equipped with a mechanic mixer and an ultraturrax homogenizer. Afterward, to increase the EO preservation on the nanoclay surface, 25 g of fumaric acid was mixed with 250 g of an EO (clove, oregano, or thymol EOs) diluted in 100 g of ethanol and then added to the mixture. The slurry was mixed for 2 h and vacuum filtered with a Büchner funnel and then dried at 80 °C for 10 h. The modified clay was then ground and sieved (100 μm) to obtain the BNT–EO AdMa powder with approximately 10 wt % of EO. The list of the AdMa obtained through the synthesis is displayed in Table 1.

**Table 1:** Synthesized Bentonite Nanoclay–Essential Oils (BNT–EO) AdMa

description	acronym
bentonite loaded with oregano oil	BNT–O
bentonite loaded with fumaric acid and oregano oil	BNT–FO
bentonite loaded with fumaric acid and thymol	BNT–FT
bentonite loaded with fumaric acid and clove oil	BNT–FC

### 2.2 Nanoclays–EO AdMa Chemical Characterization

Molecular interactions between BNT and the different EOs selected were investigated through a Thermo Nicolet Nexus 670 Fourier-transform infrared (FTIR) spectrophotometer equipped with a Smart Orbit Single Reflection Diamond ATR (attenuated total reflection) accessory, from 4000 to 400 cm^–1^ for 64 scans with 4 cm^–1^ resolution. The weight variation of the AdMa as a function of temperature and time in a controlled atmosphere was measured by thermogravimetric analysis (TGA) using a Q50 instrument (TA Instruments). The temperature ranged from 30 to 900 °C with a heating rate of 10 °C·min^–1^. Each AdMa (approximately 30 mg) was placed in platinum crucibles, and measurements were performed under a dynamic air atmosphere. Origin 8.5 software was used for data elaboration.

The amount of the different volatile compounds was determined by gas chromatography coupled with mass spectrometry (GC-MS). For the extraction of volatiles, approximately 120 mg of each AdMa was placed in a sealed flask with 10 mL of HPLC-grade acetone, stirred continuously for 2 h, and subsequently sonicated for 5 min. The resulting extract was diluted 10-fold prior to chromatographic analysis. Calibration curves were obtained using analytical standards of carvacrol and thymol (Merck). Analyses were carried out through an EVOQ GCTQ PREM EI&CI system equipped with a 436GC gas chromatograph and a COMBI-PAL autosampler coupled to a triple quadrupole mass spectrometer. Separation was achieved using an Rxi-5Sil MS column (30 m × 0.25 mm, 0.25 μm film thickness). The oven program started at 50 °C (5 min hold), ramped to 300 °C at 25 °C·min^–1^, and was maintained at the final temperature for 18 min. Helium was used as the carrier gas at 1 mL·min^–1^. Injector and detector temperatures were set at 250 °C. One microliter of each sample (0.1% in acetone) was injected in splitless mode. Mass spectra were recorded in electron impact (EI) mode at 70 eV, with a mass range of 30–850 Da. The source and transfer line were maintained at 250 °C, with a solvent delay of 4 min. Compound identification was performed by comparing retention times and mass spectra with those of reference standards and the Wiley mass spectral library.

### 2.3 *E. amylovora* Culture Conditions

*E. amylovora* Ea110 was stored in 15% glycerol at −80 °C. For each assay, the strain was inoculated into Luria–Bertani broth (LB) and incubated overnight at 28 °C with shaking at 200 rpm. The overnight culture was then diluted in fresh LB to prepare the bacterial inoculum for either *in vitro* or field trials.

### 2.4 *In Vitro* Antimicrobial Assay and Field Trial Setup

The antimicrobial *in vitro* activity of each AdMa listed in Table 1 was evaluated by incubating *E. amylovora* Ea110 in LB broth and quantifying the total viable *E. amylovora* colonies after a 24 h incubation period. Briefly, 80 μL of bacterial inoculum was added to each well of a 96-well plate, followed by 120 μL of test formulation diluted in LB, to obtain a final volume of 200 μL and a final bacterial concentration of 2·10^6^ CFU·mL^–1^. Different concentrations of the AdMa, 0.5 and 5 g·L^–1^, were added to the LB broth, with three biological replicates per treatment. Streptomycin and nontreated LB broth were included as positive and negative controls. The inoculated *E. amylovora* culture was maintained at 28 °C for 24 h with shaking. OD_600_ readings were taken by an iMark Microplate Absorbance Reader (BioRad Laboratories, Inc.) at 30 min intervals. At the end of the incubation, 30 μL from each well was plated on an LB agar plate and incubated at 28 °C overnight. Bacterial colonies on each plate were counted.

To assess the ability of BNT–EO formulations in reducing fire blight under field conditions, apple trees inoculated with the pathogen were treated with different EO and BNT–EO formulations listed in Table S1. Field trials were performed at the Connecticut Agricultural Experiment Station (CAES) Lockwood Farm in Hamden (CT, USA), in May 2023 and 2024. Fifty-six-year-old apple trees of the cultivar “golden smoothie” were used in this study. Anticipating potential efficacy variation due to differing environmental conditions, the concentration used in the field was increased to 10 g·L^–1^ for BNT, oregano oil (O-Oil), BNT–O, and BNT–FO, while Thyme Guard (T-G) and streptomycin were tested at 0.5 wt % in water (as suggested by the vendor) and at 0.1 g·L^–1^, respectively. Treatments were applied twice, once at 90% bloom (90% of flowers have their petals open at the time of application), and again at petal fall, approximately 36 h after the first application. *E. amylovora* strain Ea110 was inoculated at full bloom, approximately 4 h after the first antimicrobial treatment and 32 h before the second antimicrobial treatment, on May 3–4 2023 and May 6–7 2024, respectively. Three biological replicates (large sections of trees) with each section containing more than 200 flower clusters were used in this study. All materials were applied using a motorized Solo backsprayer Model 433 to the entire tree until dripping.

### 2.5 Disease Assessment and qPCR-Based Pathogen Quantification

Blossom blight reduction efficacy was evaluated by quantifying the percentage of flower clusters that display the blossom blight symptoms in the total flower clusters.^30^ Disease symptoms were assessed 2 weeks after treatment, on the following dates: (i) May 19, 2023 (treatment on May 3–4, 2023); (ii) May 28, 2024 (treatment on May 6–7, 2024).

Furthermore, the abundance of *E. amylovora* on collected flower samples was quantified by determining the cycle threshold (CT) value of the *E. amylovora*-specific gene *amsC* ^31^ and the apple tubulin gene,^10^ as described in a previous published research.^32^ Flowers were collected 4 days after the treatments (i.e., 5 days after Ea inoculation). Petals were removed from a flower, and the remaining part of the flower was placed in an individual Eppendorf tube with 500 μL of sterile water. The tube containing the flower and sterile water was vortexed for 30 s at maximized speed, followed by a water bath sonication for 3 min. The flower wash was centrifuged at 10,000 rpm for 5 min to collect the cell pellets, which were then used for RNA isolation using the RNeasy kit (Qiagen, MD, USA). qPCR of the *amsC* gene was performed using a SsoAdvanced universal SYBR Green supermix (BioRad, CA, USA). The relative abundance of *E. amylovora* was determined using the ΔΔ*C*_t_ method.^33^ Briefly, amsC gene copy numbers were normalized to apple tubulin gene copy numbers (endogenous control),^10^ and the resulting values were expressed relative to the inoculated, nontreated control group.

### 2.6 Inorganic Element Quantification after Field Treatment

The presence of typical inorganic elements belonging to the materials used for field trials was investigated in at least three biologically independent replicates, harvesting each stigma 2 weeks after the treatment. Briefly, each stigma was oven-dried at 40 °C overnight. Then, ∼0.2 g of each stigma was acid digested with 3 mL HNO_3_ 65% w/w and 1 mL H_2_O_2_ 30% w/w at 115 °C using a hot plate for 45 min; the final volume was adjusted to 25 mL and then analyzed by Inductively Coupled Plasma–Optical Emission Spectrometry (ICP-OES) (iCAP Pro XP, Thermo Fisher Scientific; Waltham, MA). To corroborate the validity of our analysis, yttrium (Y) was used as an internal standard for every sample, and a Standard Reference Material (SRM, NIST 1573a) was included in the analysis.

### 2.7 Statistical Data Analysis

Data on *E. amylovora* (Ea110) flower infection on stigmas were analyzed using an Aligned Rank Transform (ART) two-way analysis of variance (ANOVA). This approach was selected because preliminary diagnostic tests indicated that the assumptions required for a classical parametric two-way ANOVA—specifically normality of residuals and homogeneity of variances—were violated.

The ART method is a nonparametric (i.e., data are not normally distributed) procedure that enables factorial ANOVA designs, including interaction terms, while avoiding distributional assumptions. Briefly, ART works by first aligning the response variable with respect to each main effect and interaction term, effectively removing the influence of the other factors. The aligned values are then converted to ranks, and a standard ANOVA is performed on these ranks. This procedure allows valid hypothesis testing of main effects and interactions using familiar ANOVA frameworks while maintaining robustness to nonnormality.^34^

The experimental factors considered were “treatment”, representing the different treatments applied to flowers, and “year”, i.e., 2023 or 2024. The interaction between treatment and year was explicitly tested to assess whether treatment effects differed between years. ART ANOVA was implemented in the R package “ARTool”, version 0.11.2, using linear models applied to aligned and ranked responses, following the procedure described by Wobbrock et al.^34^ Type II sums of squares were used to evaluate main effects and interactions. Statistical significance was assessed at α < 0.05. Only treatments common to both field seasons were included in the two-year ART ANOVA and associated post hoc comparisons. Treatments evaluated in only one year were excluded from this analysis.

Relative *E. amylovora* abundance data obtained by qPCR were analyzed separately for the 2023 field trial. Because relative abundance values were right-skewed, data were log-transformed prior to analysis using log_10_(relative abundance + 10^–6^). Treatment effects were evaluated by one-way ANOVA, followed by Tukey’s honestly significant difference test for post hoc pairwise comparisons using the *aov* and *TukeyHSD* functions in R. Differences were considered significant at α < 0.05

Lastly, mean elemental concentrations (±SD) of inorganics obtained by ICP-OES were statistically analyzed by comparing BNT–O with the certified NIST SRM 1573a reference, using two-tailed one-sample *t* tests, with significance determined at α = 0.05. Recovery (%) was also calculated by comparing measured elemental concentrations in BNT–O-treated samples to certified NIST SRM 1573a reference values.

## 3 Results

### 3.1 Physicochemical Characterization of BNT–EO AdMa

The physicochemical characterization of pristine BNT was published in Brunelli et al., 2025, and a summary of the key information is displayed in Table S2. The encapsulation of clove EO onto the BNT surface was demonstrated through Fourier-Transform Infrared (FTIR) spectroscopy and thermogravimetric analysis (TGA). Moreover, the same analytical techniques confirmed the encapsulation of thymol and oregano onto BNT (Figures 1 and 2).

**Figure 1:**
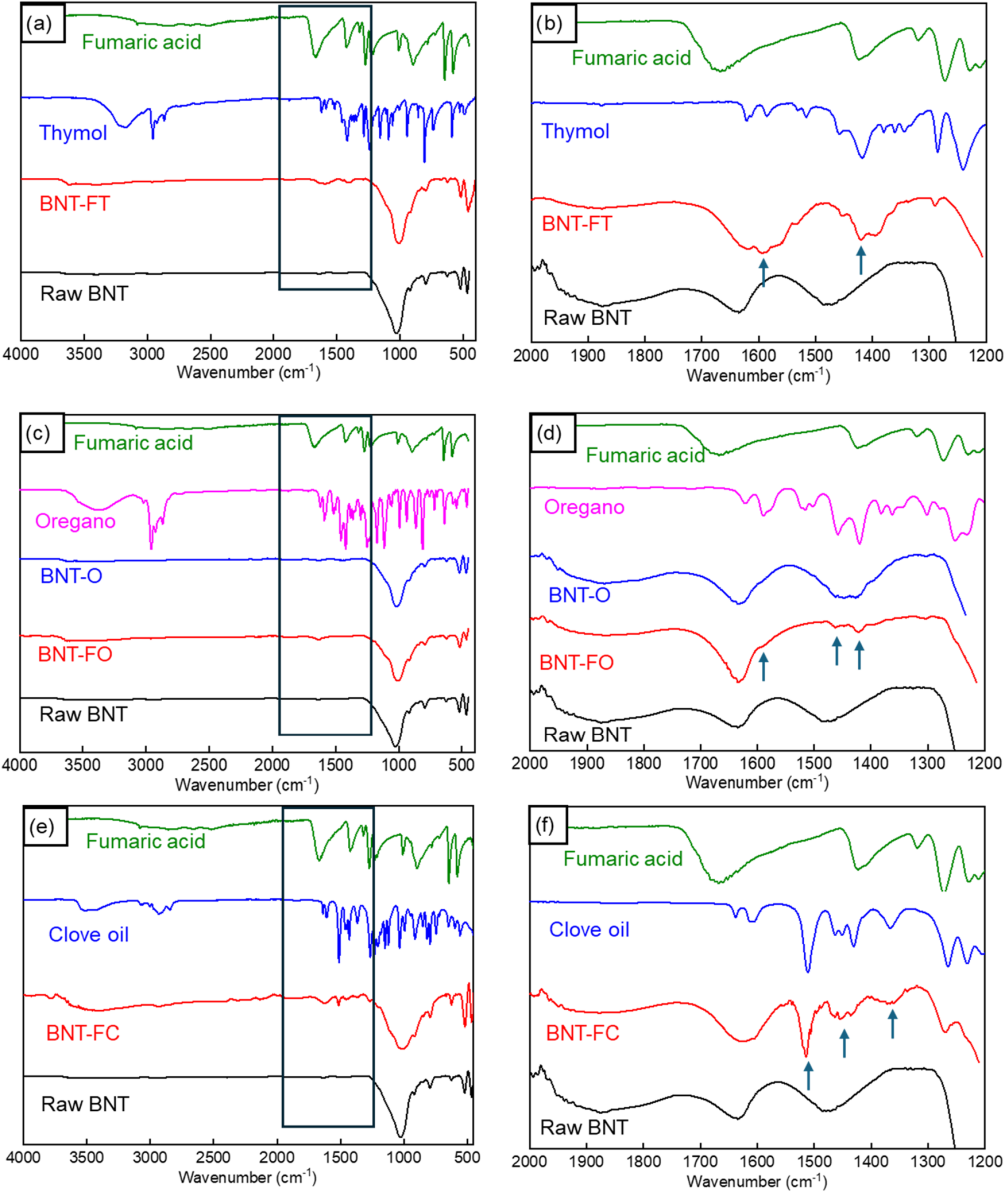
FTIR spectra of raw and modified BNT–EO advanced materials and reference compounds. (a) Full FTIR spectra of the thymol series, including fumaric acid, thymol, and BNT–FT; (b) expanded 2000–1200 cm^–1^ region of the thymol series. (c) Full FTIR spectra of the oregano oil series, including fumaric acid, oregano oil, BNT–O, and BNT–FO; (d) expanded 2000–1200 cm^–1^ region of the oregano oil series. (e) Full FTIR spectra of the clove oil series, including fumaric acid, clove oil, and BNT–FC; (f) expanded 2000–1200 cm^–1^ region of the clove oil series. Highlighted boxes in panels (a, c, e) indicate the spectral regions enlarged in panels (b, d, f), respectively. Arrows indicate representative bands associated with EO- and/or fumaric acid-related functional groups in the BNT–EO formulations.

**Figure 2:**
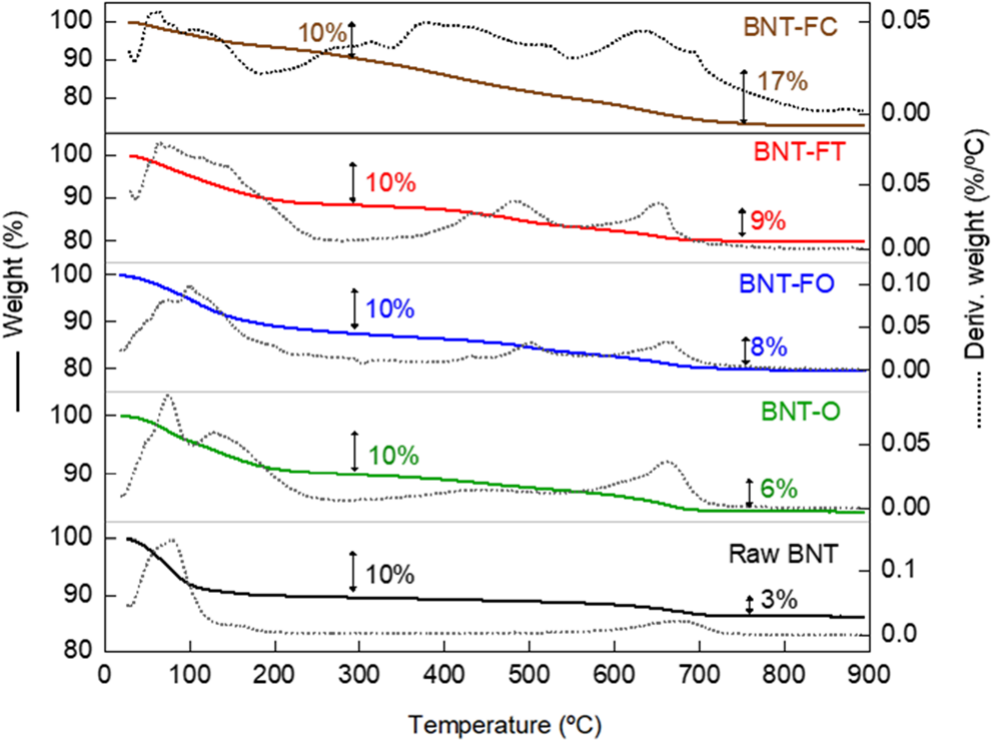
TGA weight loss (continuous line) and derivative thermogravimetry (DTG) (dashed line) curves of raw BNT, BNT–FT, BNT–O, and BNT–FO.

The FTIR spectra (Figure 1) demonstrate the successful incorporation of EO onto bentonite. Raw bentonite shows an intense Si–O stretching bands that arise around 1030–1040 cm^–1^, and a characteristic band at 1637 cm^–1^ (H_2_O bending) that appears in all samples,^35^ while the band near 1470 cm^–1^ is assigned in the literature to clay-bound COO^–^ groups.^36^ Each modified clay exhibits the main characteristic bands of both the clay and the added organic components: O–H and Si–O from bentonite, C–H and aromatic C=C from thymol/oregano, and COO^–^ from fumaric acid. The presence of EO signals (thymol or oregano) in the modified clays is confirmed by the matching bands (e.g., aromatic ring at 1610–1620 cm^–1^ and methyl/methylene bending at 1460 cm^–1^) with those of the pure oils.^36,37^ Specific bands between 1500 and 1300 cm^–1^, such as the 1460 cm^–1^ (aliphatic −CH_2_ scissoring and −CH_3_ asymmetric bending) and 1460–1420 cm^–1^ (−CH_3_ symmetric bending and C–O–H in-plane bend), are highlighted with arrows in the expanded plots (b) and (d), further confirming the presence of organic groups on the clay.

In Figure 2, the TGA and derivative thermogravimetry (DTG) curves of the BNT-based samples (raw BNT, BNT–O, BNT–FO, BNT–FT, and BNT–FC) reveal clear differences in their thermal behavior. Raw BNT exhibited only the typical dehydration (DTG peak at 77 °C) and dehydroxylation steps at 680 °C, while BNT–EO-based samples exhibit a noticeable shoulder in the DTG curve above 100 °C, which is attributed to the presence of the EOs. The DTG profiles also show distinct degradation peaks for the synthesized AdMa, with a broad peak area for BNT–FC starting from around 300 °C and around 430 and 480 °C for the other fumaric acid-based AdMa, indicating the major thermal decomposition steps of the acid component. Furthermore, the sample containing oregano but no fumaric acid (BNT–O) shows two main DTG peaks at approximately 75 and 130 °C, which can be ascribed to the evaporation and thermal degradation of water and the EO. By contrast, the sample containing both fumaric acid and oregano (BNT–FO) displays a dominant DTG peak at around 133 °C, together with a shoulder near 100 °C. The attenuation of the low-temperature mass-loss event and the shift toward a higher dominant degradation temperature in BNT–FO suggest a partial thermal stabilization of oregano components in the presence of fumaric acid.

GC-MS analysis in Table S3 shows that the composition of clays loaded with thymol or oregano differed substantially from the pure standards. In the thymol-loaded sample (BNT–FT), thymol constituted approximately 95% of the total organic composition, whereas carvacrol accounted for only 2.4%. By contrast, in the oregano-loaded clay samples, the relative abundance of carvacrol increased substantially, from 73% in the original oregano to 91–93% in BNT–FO and BNT–O, while thymol reached 3–6%. These findings indicate that the clay matrix, under thermal treatment, actively induces chemical transformations during the EO-loading process. Furthermore, GC-MS analysis detected thymoquinone in all BNT-based samples at concentrations ranging from 0.2 to 2.3%. This compound was not observed in either the pure oregano or thymol reference standards, suggesting the formation of oxidative byproducts during the processing of the clay composites. As expected, thymoquinone formation occurs in all samples due to heat-induced oxidation during drying. However, a lower relative abundance of thymoquinone was observed in the fumaric acid-treated sample (BNT–FO) compared to BNT–O, indicating a mitigating effect of fumaric acid on oxidation reactions. Specifically, the inclusion of fumaric acid effectively reduced the formation of thymoquinone and helped maintain a more stable thymol/carvacrol ratio. This strongly suggests a stabilizing role of the organic acid when added to clays. In contrast, the clove-oil-loaded sample (BNT–FC) exhibited a markedly different behavior. The analysis revealed an almost exclusive retention of eugenol (99.97%), while the relative content of caryophyllene decreased sharply from ∼16% in the pure oil to only 0.03% after loading. This selective retention can be attributed to stronger interactions of eugenol with the BNT matrix, likely involving hydrogen bonding and polar interactions with surface hydroxyls and interlayer sites, whereas the more volatile and less polar caryophyllene was largely lost during drying.

Furthermore, several minor and more volatile constituents typical of oregano, such as α-pinene, myrcene, linalool, and terpineol, were lost or drastically reduced upon loading, most likely due to their higher volatility and poor retention in the clay matrix. Only small traces of borneol, eugenol, and caryophyllene were detected, confirming that the clay preferentially retains the less volatile phenolic monoterpenes. Overall, the results indicate that loading onto BNT promotes isomerization and oxidation of the phenolic fraction, while fumaric acid mitigates these transformations, and the matrix selectively retains the more stable, less volatile constituents.

### 3.2 *In Vitro* Antimicrobial Activity of BNT–EO AdMa against Ea110

Compared with the nontreated control, supplementation with streptomycin effectively inhibited bacterial growth (Figure 3). In contrast, oregano alone did not exhibit antimicrobial activity against *E. amylovora* at either 0.5 g·L^–1^ or 5 g·L^–1^. Notably, Figure 3 also shows that supplementation with the BNT–O formulation resulted in complete inhibition of *E. amylovora* growth at 5 g·L^–1^. BNT alone showed no antimicrobial effect at either concentration, indicating that the enhanced antimicrobial activity is attributable to the BNT–EO formulation rather than the BNT nanoclay itself. Based on this assay, the minimum inhibitory concentration (MIC) of the different BNT–EO AdMa was estimated at 5 g·L^–1^. To estimate the minimum inhibitory concentration (MIC) for BNT–O and oregano oil, additional assays were conducted using intermediate concentrations (2, 3, and 4 g·L^–1^ for BNT–O and 1, 3, 5, and 10 g·L^–1^ for oregano oil), which showed an MIC of 5 g·L^–1^ (Table S4).

**Figure 3:**
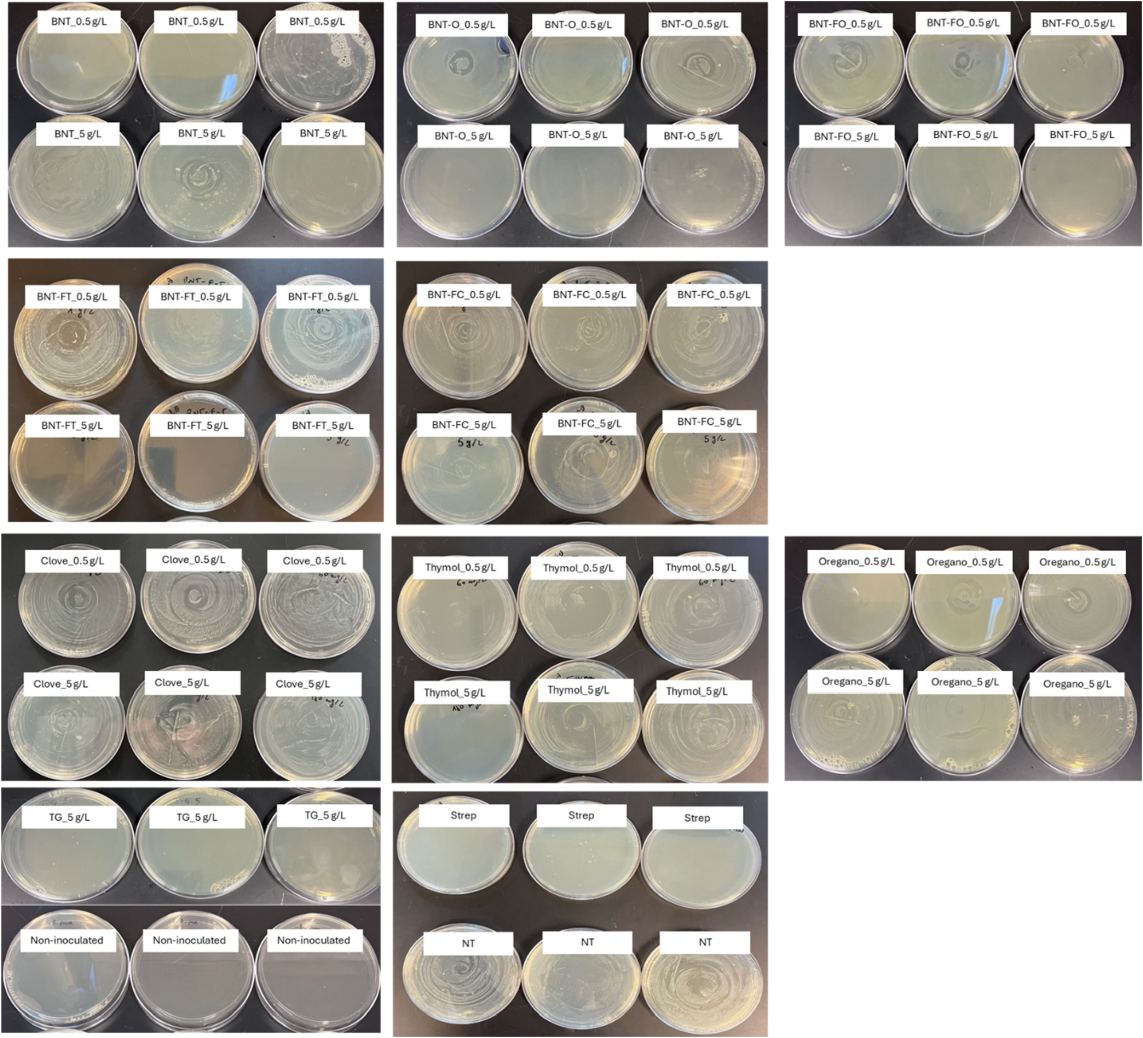
Growth of Ea110 in the presence of BNT, BNT–O, BNT–FO, BNT–FT, BNT–FC, all essential oils (EOs), and Thyme Guard (T-G), at 0.5 and 5 g·L^–1^. Streptomycin was tested at 50 mg·L^–1^. Noninoculated refers to LB only; NT means only Ea110, but without any BNT–EO formulation treatment.

### 3.3 Field Evaluation of BNT–EO AdMa against Fire Blight

#### 3.3.1 Disease Control Efficacy and Pathogen Abundance in Treated Flowers

Flower infection data are shown in Figure 4. In 2023, streptomycin significantly reduced fire blight incidence to 11% infected flowers, compared with 58% in the nontreated control (Figure 4A). Treatments with EO alone (clove, oregano, and thyme) or with nanoclay BNT alone resulted in only a slight reduction in disease incidence (Figure 4A). In contrast, BNT–EO formulations—although containing the same amount of EO as their corresponding oil-only treatments—consistently enhanced disease reduction (Figure 4A). Among these, the BNT–O treatment was the most effective (14% flower infection), a level comparable to that of streptomycin. In 2024, overall disease incidence increased substantially across treatments (Figure 4B). While BNT–O again provided greater infection reduction than oregano alone or BNT alone, its efficacy declined relative to 2023. Notably, BNT–FO also showed promising results, suggesting that formulations embedding carvacrol may provide stronger antibacterial activity than those containing thymol or eugenol. However, the difference between streptomycin and all other materials became more pronounced in 2024, indicating a widening performance gap under the higher disease pressure observed in that season. We only evaluated the blossom blight stage of infection. Future evaluations should assess fire blight suppression throughout the entire growing season, including the shoot blight stage.

**Figure 4:**
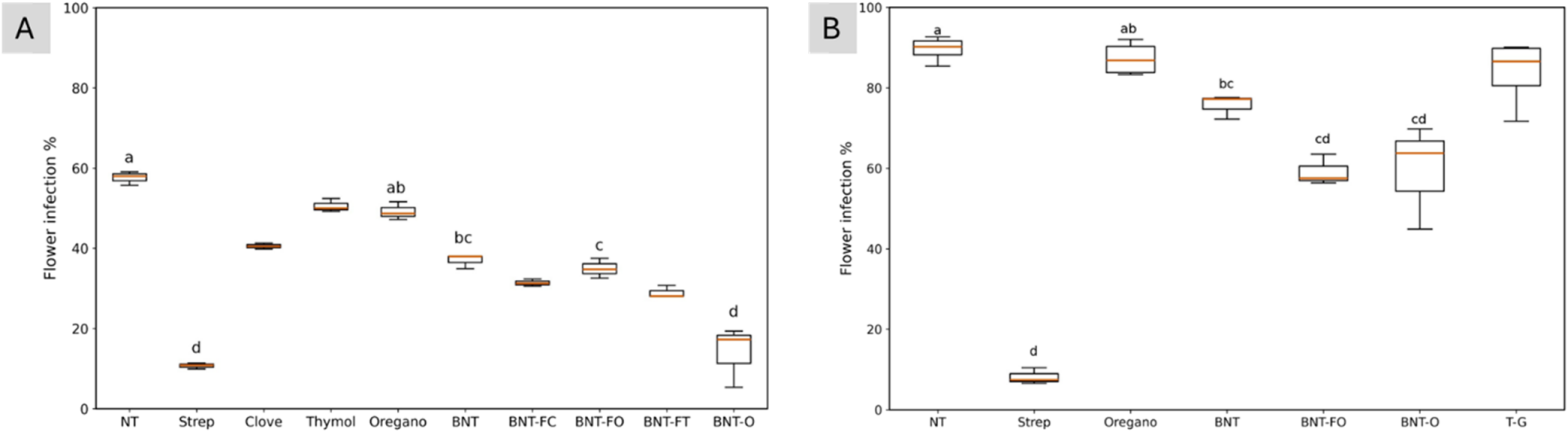
Flower infection after treating apple trees with the BNT–EO AdMas during (A) spring 2023 and (B) spring 2024. NT: Ea110 only, without any treatment, strep: streptomycin, clove: clove oil, thymo: thymol oil, oregano: oregano oil, BNT: bentonite, BNT–FC: bentonite with fumaric acid and clove oil, BNT–FO: bentonite with fumaric acid and oregano oil, BNT–FT: bentonite with fumaric acid and thymol oil, BNT–O: bentonite with oregano oil, and T-G: Thyme Guard. Boxplots show the median as the horizontal line within the box, the interquartile range as the box, and the whiskers as the minimum and maximum values. Each treatment included three biological replicates. Different letters indicate significant differences among treatments within the same year based on Tukey-adjusted post hoc comparisons following ART ANOVA (α < 0.05). Treatments sharing at least one letter are not significantly different. Treatments present in only one year were not included in the two-year ART post hoc comparison.

The ART ANOVA, conducted on flower infection data for treatments common to both years, was applied to determine (i) whether treatments differed in efficacy, (ii) whether the overall disease pressure differed between years, and (iii) whether treatment effects varied between years. The results are summarized in Table 2.

**Table 2:** ART ANOVA Results, Specifying the Degrees of Freedom (df), *F*- and *p*-Values

effect	df	*F*-value	*p*-value
treatment	5	99.24	4.3·10^–16^
year (2023 vs 2024)	1	80.00	2.0·10^–9^
treatment × year interaction	5	9.808	2.3·10^–5^

ART ANOVA revealed significant effects of treatment, year, and their interaction (*p* < 0.001 for all effects), indicating that flower infection differed significantly among treatments, overall disease levels differed between 2023 and 2024, and treatment efficacy was influenced by the field season. The significant treatment × year interaction further indicates that treatment rankings and/or effect sizes were not fully consistent across the two field seasons, suggesting sensitivity to interannual environmental variability. Flower infection was significantly affected by treatment, year, and their interaction (Figure 4 and Table 2), indicating that treatment efficacy differed between field seasons. Post hoc comparisons were therefore conducted within each year for treatments common to both seasons; treatments evaluated in only one year were excluded from this two-year ART analysis. Results are reported in Table S5 and shown as compact letter displays in Figure 4. In 2023, BNT–O and streptomycin produced the lowest infection levels and were statistically comparable, with both treatments significantly reducing infection relative to the nontreated (NT) control. BNT–FO and BNT also reduced infection relative to the NT control, whereas oregano alone did not. In 2024, streptomycin provided the greatest disease reduction and differed significantly from all other common treatments. BNT–O and BNT–FO produced intermediate infection levels and remained significantly different from both the NT control and streptomycin, whereas BNT and oregano did not differ from the NT control. These results indicate that BNT–EO formulations, particularly BNT–O and BNT–FO, can decrease flower infection, but their efficacy can be strongly influenced by the field season.

To determine whether reduced flower infection was associated with lower *E. amylovora* abundance, pathogen populations were quantified in floral samples by qPCR (Figure 5). This analysis focused on the 2023 field season, where treatment effects on pathogen abundance were more clearly differentiated than those in 2024.

**Figure 5:**
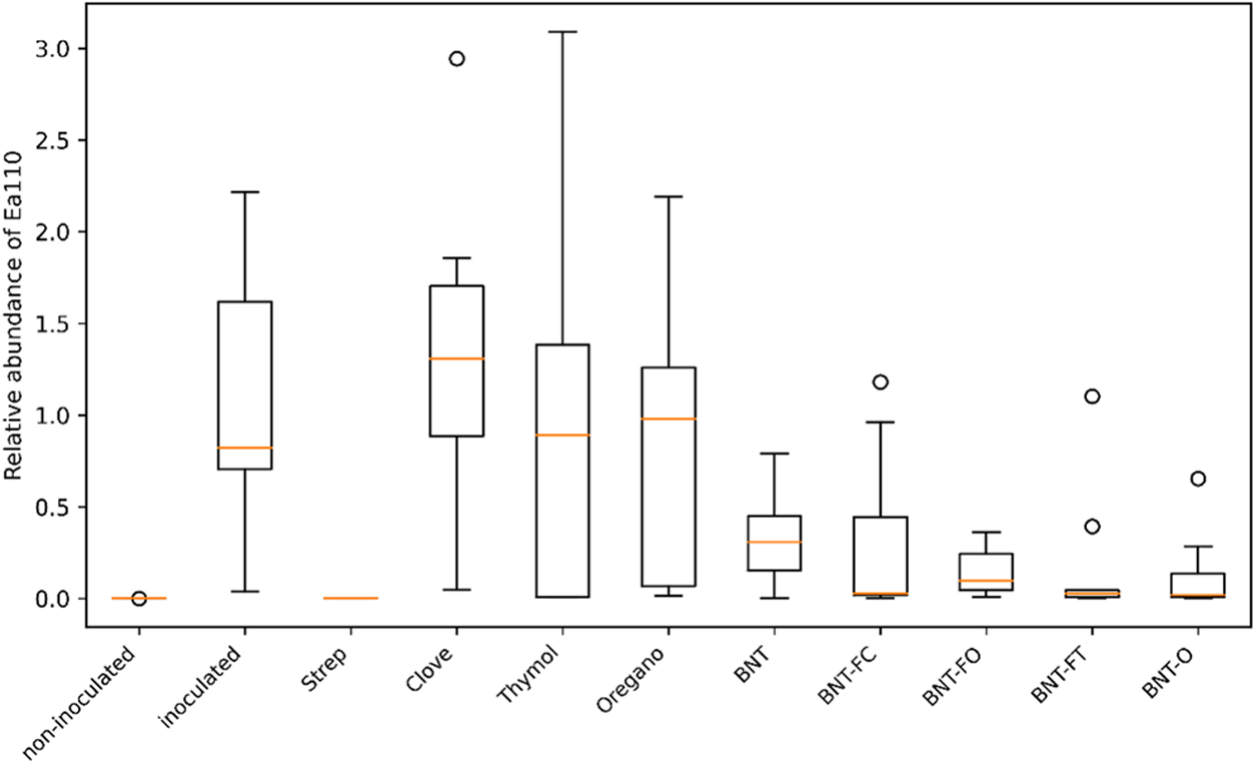
Relative abundance of Ea (amsC copy number) using quantitative PCR (qPCR) data from flowers collected 4 days after the treatments (5 days after Ea inoculation) in the 2023 field trial. Noninoculated: no treatments at all, inoculated: Ea110 only, strep: streptomycin, clove: clove oil; thymo: thymol oil, oregano: oregano oil, BNT: bentonite, BNT–FC: bentonite with fumaric acid and clove oil, BNT–FO: bentonite with fumaric acid and oregano oil, BNT–FT: bentonite with fumaric acid and thymol oil, and BNT–O: bentonite with oregano oil. The relative *E. amylovora* abundance was determined by measuring Ea *amsC* copy number and the apple tubulin gene copy number, and was compared to the inoculated but the nontreated group using the ΔΔ*C*_t_ method. Boxplots show the median as the horizontal line within the box, the interquartile range as the box, and whiskers as values within 1.5× the interquartile range. Dots represent outliers beyond the whiskers. Each treatment included nine biological replicates, except the noninoculated and streptomycin treatments, which included eight and seven biological replicates, respectively.

Treatment significantly affected log_10_-transformed relative *E. amylovora* abundance, and Tukey HSD post hoc comparisons were used to identify differences among treatments (Table S6). Consistent with the flower infection results, EO-only treatments and BNT alone were not significantly different from the inoculated, nontreated control, indicating limited effects on *E. amylovora* populations when applied alone. In contrast, streptomycin and selected EO-loaded nanoclay formulations showed significantly lower pathogen abundance than the inoculated control. Among the BNT–EO formulations, BNT–O had one of the lowest median Ea110 abundance values and was statistically comparable to streptomycin, although both treatments remained higher than the noninoculated control.

Nanoclay–EO formulations, particularly BNT–O and BNT–FC, reduced the relative abundance of Ea110 by approximately 1 order of magnitude compared with EO-only or BNT-Only treatments. Median values for BNT–O were typically below 0.02, whereas EO-only treatments frequently exhibited median values near or above 1.0 and reached a maximum exceeding 2.0. Similarly, BNT–FC showed substantially lower pathogen levels than BNT alone, with values ranging from 0.15 to 0.80. Although several EO-loaded nanoclay formulations decreased the Ea110 population, BNT–O produced the greatest reduction in flower infection, suggesting that its activity may involve mechanisms beyond direct pathogen inhibition, such as improved persistence, adhesion, or coverage on floral tissues. Because these formulation-related effects may be influenced by field conditions, environmental covariates recorded during the study are reported in Table 3 to provide context for the observed variation in *E. amylovora* flower infection and relative abundance.

**Table 3:** Environmental Parameters Recorded at Lockwood Farm (Hamden, CT, USA) during the Bloom Periods (April–May) of 2023 and 2024^a^

	temperature (°C)				rain (mm)	relative humidity (RH) (%)	mean wind (m·s^–1^)	prevailing wind
year (April–May)	mean	min	max	median				
2023	15.0–16.5	8.0–9.5	22.0–24.5	15.5–16.0	115–125	55–60	3.5–4.5	SW–W
2024	13.0–14.5	6.5–8.0	19.5–21.5	13.5–14.0	75–90	42–48	4.0–5.0	N–NW/W

a Data were obtained from Lockwood Farm and NOAA records. Values are reported as ranges observed during the study period for each parameter.

Although environmental variables were not included directly in the statistical model, weather records in Table 3 can provide important context for these interannual differences. During the treatment period (April–May), conditions in 2024 were characterized by cooler median temperatures, reduced rainfall, lower relative humidity, and a greater frequency of N–NW winds compared with 2023. Such conditions may have promoted faster blossom drying and reduced treatment persistence, potentially contributing to the observed year-dependent variation in treatment efficacy.

#### 3.3.2 Residual Levels of BNT–EO AdMa on Treated Flowers

One putative advantage of the BNT–EO formulation is that the nanoclay carrier (BNT) may exhibit prolonged affinity for plant tissue after application. To test this hypothesis, apple flowers treated with BNT–O, which was the most effective in reducing flower infection by Ea110, were collected 9 days after treatment and analyzed for aluminum (Al), sodium (Na), and silicon (Si), three major elemental constituents of BNT. Elemental analysis revealed statistically significant enrichment of Al, Na, and Si in BNT–O-treated stigmas compared with both nontreated and oregano-treated samples (two-sample *t* tests, *p* < 0.001 for all elements). In contrast, flowers treated with oregano alone did not exhibit elevated concentrations of these inorganic elements relative to the nontreated control (Figure 6). These results provide quantitative evidence that nanoclay-derived mineral components persist on floral tissues for more than 1 week postapplication, encompassing the critical period for *E. amylovora* flower infection.

**Figure 6:**
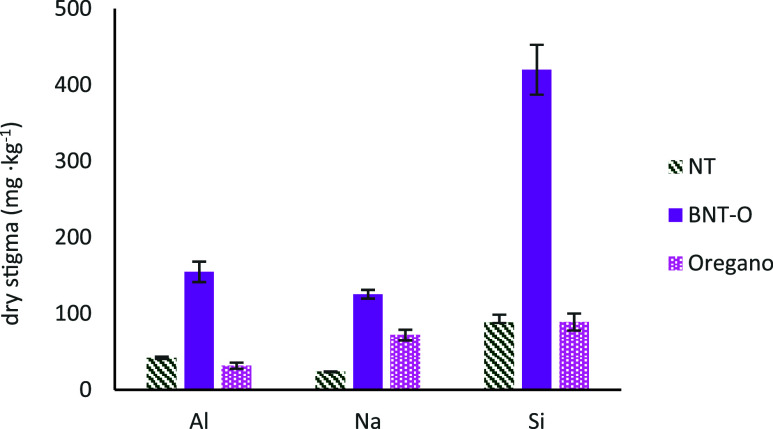
Elemental concentration of Al, Na, and Si (mg·kg^–1^ of dry stigma). The results are expressed as the means ± standard deviation of three independent samples.

Analytical accuracy was evaluated using the NIST SRM 1573a certified reference material. Elemental recovery was 85.6% for Al, 110.4% for Na, and 109.3% for Si. One-sample *t* tests comparing BNT–O measurements to certified NIST values indicated no significant deviation from the reference material (*p* > 0.05), confirming the reliability of quantification and demonstrating that the deposited mineral profile is consistent with the clay-based formulation.

## 4 Discussion

Natural BNT nanocomposites loaded with clove, thymol, or oregano EOs were synthesized and evaluated as alternative control strategies against fire blight caused by *E. amylovora*. The results demonstrate that these AdMa effectively inhibited bacterial growth under *in vitro* conditions with a higher efficacy than the EO alone. Notably, selected formulations also effectively reduced fire blight infection in apple orchards, demonstrating a strong potential as alternatives to antibiotics in plant agriculture. The results demonstrate that these AdMa effectively inhibited bacterial growth under *in vitro* conditions with a higher efficacy than the EO alone.

Overall, comparison of the performance of AdMa evaluated both *in vitro* and under field conditions indicates that carvacrol (active ingredient of oregano, present in both BNT–O and BNT–FO) exhibits higher antibacterial activity against *E. amylovora* Ea110 than thymol (thyme oil) and eugenol (main component of clove oil). As suggested in the literature,^38,39^ this enhanced efficacy can be attributed to carvacrol’s favorable molecular structure, particularly the positioning of the hydroxyl group and high lipophilicity, which facilitate strong interactions with bacterial membranes and promote membrane destabilization. Consistent with recent pathogen-specific evidence by Zhou et al.,^40^ carvacrol is able to increase cell membrane permeability, inhibit motility and biofilm formation, down-regulate virulence-associated genes, and ultimately elevate bacterial cell death. Carvacrol and thymol are positional isomers and possess a phenolic hydroxyl group, which is key to their antimicrobial activity. As reported by Radocchia et al.,^41^ both compounds disrupt membrane integrity, inhibit biofilm formation, and reduce metabolic activity, suggesting largely overlapping mechanisms of action. Differences in antibacterial potency between the two monoterpenes may be more likely attributable to slight structural variations affecting membrane partitioning and interaction with lipid bilayers rather than modes of action. Unlike carvacrol and thymol, eugenol exhibited comparatively weaker antibacterial activity against *E. amylovora* under field conditions, possibly due to differences in membrane interaction efficiency arising from its distinct structural features. Notably, the disease reduction efficacy of carvacrol is further enhanced when encapsulated in nanoclay as compared to when applied alone. The enhancement of the decrease in flower infection with nanotechnology may be explained in twofold. First, the nano encapsulation may enhance carvarcrol’s antimicrobial effect against *E. amylovora* growth, as it was demonstrated both *in vitro* and in the field. The enrichment of carvacrol during the encapsulation process likely contributes significantly to the observed efficacy. More broadly, EO loading modifies composition through selective retention, volatilization, and partial oxidation, which, together with the stabilizing effect of the clay matrix, may enhance antimicrobial performance by improving the availability and persistence of active compounds. Second, the nano encapsulation may enhance residue persistence by improving adhesion to floral surfaces after application, as evidenced by the retention of BNT on apple flowers for more than 1 week postapplication.

Although the specific mechanisms of nanoclay-based materials against *E. amylovora* remain poorly defined, studies on nanoclay systems in other bacterial models indicate that these materials can exert antimicrobial effects through multiple mechanisms, including bacterial adsorption onto high-surface-area clay particles, membrane perturbation induced by direct particle–cell interactions, and ion-mediated toxicity arising from the clay matrix.^42–45^ However, despite the extended persistence and antimicrobial activity potentially exerted by nano encapsulation, infections may still occur. This may be due to the recovery of residual bacterial populations over time or the ability of bacteria to reach nectarthodes regardless of treatment coverage,^46^ as *E. amylovora* populations on floral surfaces are highly dynamic and influenced by environmental conditions.^47^

Notably, differences in efficacy were observed between the 2023 and 2024 field trials. Such variability is expected under orchard conditions, where environmental factors—including temperature, relative humidity, rainfall, and UV exposure—can strongly influence both *E. amylovora* population dynamics and treatment performance. These conditions may affect the stability and persistence of EO components on floral surfaces, as well as the timing and severity of infection events. The higher variability observed in 2024 likely reflects a stronger environmental influence on both pathogen pressure and formulation efficacy. These findings highlight the importance of multiseason evaluations to assess robustness under diverse field conditions. This also points to future efforts to develop improved formulations to minimize the seasonal variations and improve control consistency.

Overall, this work underscores the potential of EO-based AdMa as promising tools for sustainable disease management in agriculture, particularly in efforts to reduce reliance on conventional antibiotics and mitigate the risks associated with antimicrobial resistance. The results presented here are a first demonstration of their efficacy under field conditions; however, further multiseason field experiments will be necessary to confirm their consistency and robustness across different environmental scenarios. Despite these encouraging outcomes, further investigation is required to fully elucidate the mechanisms governing their antimicrobial activity, efficacy, and performance under various environmental conditions. In particular, future studies should characterize the relative contributions of chemical mechanisms (such as the controlled release and bioactivity of EO components), physical mechanisms (including surface interactions, barrier effects, or contact-mediated disruption of bacterial cells), or synergistic combinations of both as a function of the different environmental parameters. Additionally, evaluating combinations of different antimicrobial active ingredients may reveal synergistic interactions that enhance the antibacterial efficacy in both laboratory and field settings. It is worth noting that a more comprehensive understanding of these mechanisms will be essential for optimizing formulation design, enhancing product stability and persistence under field conditions, and improving reproducibility of efficacy across different environmental and agronomic contexts. Ultimately, such knowledge will support the rational development and broader adoption of EO-based AdMa within integrated, environmentally responsible crop protection strategies that align with the goals of sustainable agriculture and reduced antibiotic use.

## Supplementary Material



## Data Availability

All data supporting this study are provided in the main text and in the Supporting Information.

## Notes

List of BNT–EO materials tested in 2023 and 2024 field trials; physicochemical properties of BNT; GC-MS composition of essential oils and BNT–EO formulations; MIC estimation for BNT–O and oregano oil; Tukey-adjusted post hoc comparisons following ART ANOVA for flower infection; and Tukey HSD pairwise comparisons for qPCR-based *E. amylovora* relative abundance (PDF)
