# Supplementary material for: Advanced Nanoencapsulated Essential Oil Materials as Novel Antimicrobials for the Control of Fire Blight in Apple
Source: J Agric Food Chem. 2026 Aug 1;74(31):24173–84. doi: 10.1021/acs.jafc.6c02932 (PMC13474928; doi:10.1021/acs.jafc.6c02932)
Supplement: Supplementary file 1 [file jf6c02932_si_001.pdf]

Supporting Information

Advanced nano-encapsulated essential oil materials as novel antimicrobials for the control of fire blight in apple

Andrea Brunelli[1*], Cástor Salgado[2], José Francisco Fernandez[2], Nubia Zuverza-Mena[3], Jason C. White[3], Quan Zeng[4*]

[1]Department of Environmental Sciences, Informatics and Statistics, Ca' Foscari University of Venice, Via Torino 155, 30170, Italy

[2]Instituto de Ceramica y Vidrio (ICV), CSIC. Kelsen 5, 28049 Madrid, Spain

[3] Department of Analytical Chemistry, The Connecticut Agricultural Experiment Station, New Haven, CT 06511, United States

[4] Department of Plant Pathology and Ecology, The Connecticut Agricultural Experiment Station, New Haven, CT 06511, United States

*Corresponding authors: andrea.brunelli@unive.it; quan.zeng@ct.gov

Table S1 – List of the different BNT-EO AdMa tested, specifying chemical composition, physical state and year of testing in field. In bold the materials tested during both campaigns (springs 2023 and 2024). After the first in-field treatment, Thyme Guard (TG) was included as a representative commercial benchmark, which is approved for organic agriculture.

**Acronym**	**Description**	**Physical state**	**1st year (spring 2023)**	**2nd year (spring 2024)**
BNT	**Bentonite**	**solid**	**X**	**X**
BNT-FC	Bentonite + Fumaric acid + Clove essential oil	solid	X	
BNT-FO	**Bentonite + Fumaric acid + Oregano essential oil**	**solid**	**X**	**X**
BNT-O	**Bentonite + Oregano essential oil**	**solid**	**X**	**X**
BNT-FT	Bentonite + Fumaric acid + Thymol	solid	X	
Clove	Clove essential oil	liquid	X	
**Oregano**	**Oregano essential oil**	**liquid**	**X**	**X**
**Thymol**	Thymol	solid	X	
TG	Thyme Guard® – Quart Concentrate	liquid		X

Table S2 – Overview of the main physico-chemical data of BNT (data from Brunelli et al.,[1]).

**Mineral composition (%)**	**Specific surface area ($m^2 \cdot g^{-1}$)**	**Total pore volume ($cm^3 \cdot g^{-1}$)**	**Cation exchange capacity ($meq \cdot 100\ g^{-1}$)**	**Carbon content (%)**
montmorillonite (82%); opal (12%); feldspar (3%); plagioclase (1%); calcite (2%)	82	0.16	86	1.8

Table S3 - Amount of EO onto BNT determined by gas chromatography coupled with mass spectrometry (GC-MS).

	Composition (%)						
**Compound (retention time, min)**	**Clove**	**Oregano**	**Thymol**	**BNT-O**	**BNT-FO**	**BNT-FC**	**BNT-FT**
**Pinene (6.49)**		0.97 ± 0.01					
**Mircene (7.25)**		1.21 ± 0.01	-	-	-		-
**p-Cymene (8.13)**		17.94 ± 0.10	-	1.2 ± 0.1	1.37 ± 0.03		0.070 ± 0.001
**Linalool (8.41)**		1.150 ± 0.001	-	-	-		-
**Borneol (9.39)**		-	-	0.30 ± 0.02	0.220 ± 0.001		-
**Terpineol (9.53)**		3.61 ± 0.02	-	0.030 ± 0.001	-		-
**Thymoquinone (9.86)**		-	-	0.52 ± 0.01	0.23 ± 0.01		2.27 ± 0.17
**Thymol (10.08)**		0.450 ± 0.001	100	6.1 ± 0.2	3.40 ± 0.02		95.07 ± 0.08
**Carvacrol (10.12)**	0.020 ± 0.001	73.17 ± 0.20	-	91.1 ± 0.1	93.55 ± 0.02		2.44 ± 0.05
**Eugenol (10.48)**	83.84 ± 0.02	0.010 ± 0.001	-	0.080 ± 0.001	0.350 ± 0.001	99.97 ± 0.01	0.06 ± 0.01
**Caryophyllene (11.76)**	15.95 ± 0.01	1.41 ± 0.01	-	0.84 ± 0.01	0.880 ± 0.001	0.030 ± 0.001	0.190 ± 0.001

Table S4 – Minimum inhibitory concentration (MIC) estimation for BNT-O and Oregano oil samples, showing the concentrations tested and corresponding microbial growth outcomes (Yes/No).

Sample (3 independent replicates)	Concentration tested ($g \cdot L^{-1}$)	Growth (Yes/No)
BNT-O	2	Yes
	3	No
	4	No
Oregano oil	1	Yes
	3	No
	5	No
	10	No

Table S5 - Tukey-adjusted post hoc pairwise comparisons within each year following ART ANOVA. Values are reported as mean ± SD. Treatments sharing at least one letter are not significantly different at $\alpha < 0.05$.

**Year**	**Treatment**	**Mean ± SD**	**Significance group**
2023	NT	0.576 ± 0.017	a
2023	Oregano	0.492 ± 0.023	ab
2023	BNT	0.370 ± 0.018	bc
2023	BNT-FO	0.350 ± 0.025	c
2023	BNT-O	0.140 ± 0.076	d
2023	Strep	0.107 ± 0.008	d
2024	NT	0.896 ± 0.032	a
2024	Oregano	0.873 ± 0.043	ab
2024	BNT	0.757 ± 0.030	bc
2024	BNT-O	0.595 ± 0.130	cd
2024	BNT-FO	0.591 ± 0.038	cd
2024	Strep	0.082 ± 0.020	d

Table S6 - Tukey HSD pairwise comparisons of log10-transformed relative abundance of *Erwinia amylovora* in apple flower samples (Figure 5). Relative abundance values were $\log_{10}$-transformed as $\log_{10}$(relative abundance + 10^{-6}) and analyzed by one-way ANOVA followed by Tukey HSD post hoc comparisons. Significant differences were determined at alpha = 0.05. Bold p-values indicate significant pairwise differences after Tukey adjustment.

Comparison	Difference	95% CI	Adjusted p-value	Significant (alpha < 0.05)
non-inoculated vs inoculated	-4.916	-6.470 to -3.362	**<0.0001**	**Yes**
non-inoculated vs Clove	-5.106	-6.660 to -3.552	**<0.0001**	**Yes**
non-inoculated vs BNT	-4.146	-5.700 to -2.592	**<0.0001**	**Yes**
non-inoculated vs BNT-FC	-3.901	-5.455 to -2.347	**<0.0001**	**Yes**
non-inoculated vs BNT-FO	-4.081	-5.635 to -2.527	**<0.0001**	**Yes**
non-inoculated vs BNT-FT	-3.515	-5.069 to -1.961	**<0.0001**	**Yes**
non-inoculated vs BNT-O	-3.336	-4.890 to -1.782	**<0.0001**	**Yes**
inoculated vs Clove	-0.190	-1.644 to 1.263	1.0000	No
inoculated vs BNT	0.770	-0.684 to 2.224	0.8034	No
inoculated vs BNT-FC	1.015	-0.439 to 2.469	0.4369	No
inoculated vs BNT-FO	0.835	-0.619 to 2.289	0.7151	No
inoculated vs BNT-FT	1.401	-0.053 to 2.855	0.0691	No
inoculated vs BNT-O	1.580	0.126 to 3.033	**0.0219**	**Yes**
Strep vs non-inoculated	2.241	0.593 to 3.890	**0.0010**	**Yes**
Strep vs inoculated	-2.674	-4.228 to -1.120	**<0.0001**	**Yes**
Strep vs Clove	-2.865	-4.419 to -1.311	**<0.0001**	**Yes**
Strep vs Oregano	-2.377	-3.931 to -0.823	**0.0001**	**Yes**
Strep vs BNT	-1.904	-3.458 to -0.350	**0.0050**	**Yes**
Strep vs BNT-FC	-1.660	-3.214 to -0.105	**0.0263**	**Yes**
Strep vs BNT-FO	-1.840	-3.394 to -0.285	**0.0080**	**Yes**
Strep vs BNT-FT	-1.274	-2.828 to 0.280	0.2125	No
Strep vs BNT-O	-1.095	-2.649 to 0.459	0.4231	No
Clove vs BNT	0.960	-0.493 to 2.414	0.5208	No
Clove vs BNT-FC	1.205	-0.248 to 2.659	0.1988	No
Clove vs BNT-FO	1.025	-0.428 to 2.479	0.4213	No
Clove vs BNT-FT	1.591	0.138 to 3.045	**0.0202**	**Yes**
Clove vs BNT-O	1.770	0.316 to 3.224	**0.0055**	**Yes**
Thymol vs non-inoculated	4.390	2.836 to 5.945	**<0.0001**	**Yes**
Thymol vs inoculated	-0.525	-1.979 to 0.928	0.9813	No
Thymol vs Strep	2.149	0.595 to 3.703	**0.0008**	**Yes**
Thymol vs Clove	-0.716	-2.169 to 0.738	0.8652	No
Thymol vs Oregano	-0.228	-1.682 to 1.226	1.0000	No
Thymol vs BNT	0.245	-1.209 to 1.698	1.0000	No
Thymol vs BNT-FC	0.490	-0.964 to 1.943	0.9889	No
Thymol vs BNT-FO	0.310	-1.144 to 1.763	0.9998	No
Thymol vs BNT-FT	0.876	-0.578 to 2.329	0.6541	No
Thymol vs BNT-O	1.054	-0.399 to 2.508	0.3793	No
Oregano vs non-inoculated	4.618	3.064 to 6.173	**<0.0001**	**Yes**
Oregano vs inoculated	-0.297	-1.751 to 1.156	0.9998	No
Oregano vs Clove	-0.488	-1.941 to 0.966	0.9892	No
Oregano vs BNT	0.473	-0.981 to 1.926	0.9915	No
Oregano vs BNT-FC	0.718	-0.736 to 2.171	0.8633	No
Oregano vs BNT-FO	0.538	-0.916 to 1.991	0.9779	No

Oregano vs BNT-FT	1.104	-0.350 to 2.557	0.3128	No
Oregano vs BNT-O	1.282	-0.171 to 2.736	0.1348	No
BNT-FC vs BNT	-0.245	-1.699 to 1.209	1.0000	No
BNT-FO vs BNT	-0.065	-1.519 to 1.389	1.0000	No
BNT-FO vs BNT-FC	0.180	-1.274 to 1.634	1.0000	No
BNT-FT vs BNT	-0.631	-2.084 to 0.823	0.9355	No
BNT-FT vs BNT-FC	-0.386	-1.840 to 1.068	0.9984	No
BNT-FT vs BNT-FO	-0.566	-2.020 to 0.888	0.9684	No
BNT-O vs BNT	-0.810	-2.263 to 0.644	0.7512	No
BNT-O vs BNT-FC	-0.565	-2.018 to 0.889	0.9689	No
BNT-O vs BNT-FO	-0.745	-2.198 to 0.709	0.8338	No
BNT-O vs BNT-FT	-0.179	-1.632 to 1.275	1.0000	No

References

(1) Brunelli, A.; Trabucco, S.; Salgado, C.; Reinosa, J. J.; Fernandez, J. F.; Serrano-Lotina, A.; Bañares, M. A.; Blosi, M.; Peijnenburg, W.; Soeteman-Hernandez, L. G.; Cassee, F. R.; Fernandes, T.; Saccardo, A.; Doak, S. H.; Fito, C.; Fernandez, E. G.; Hermosilla, J. S.; Aguirre, I. G.; Rauscher, H.; Stone, V.; Moschini, E.; Livieri, A.; Pizzol, L.; Hristozov, D.; Marcomini, A.; Badetti, E. Safe and Sustainable by Design-Compliant LDPE Food Packaging Embedding Multicomponent Nanomaterials for Food Protection. *Env. Sci Nano* **2025**, *12* (12), 5489–5504. https://doi.org/10.1039/D5EN00435G.
